# The Unity of Medicine, an Introductory Lecture, Delivered October 14th, 1856

**Published:** 1857-02

**Authors:** 


*The Unity of Medicine, an Introductory Lecture, delivered October* 14*th*, 1856. By ALFRED STILLE, M.D. and Prof. of Theory and Practice of Medicine.

This is a well-written and beautifully-printed lecture, abounding in classic allusions and poetic illustrations.

